# Primary breast lymphoma: a case series and review of the literature

**DOI:** 10.1186/s13256-023-03998-8

**Published:** 2023-06-28

**Authors:** S. Sakhri, M. Aloui, M. Bouhani, H. Bouaziz, S. Kamoun, M. Slimene, T. Ben Dhieb

**Affiliations:** 1 Surgical Oncology Department, Salah Azaiez Institute of Oncology, Tunis, Tunisia; 2 Pathology Department, Salah Azaiez Institute of Oncology, Tunis, Tunisia

**Keywords:** Breast cancer, Non-Hodgkin’s lymphoma, Primary breast lymphoma, Treatment

## Abstract

**Background:**

Primary breast lymphoma (PBL) is a very rare form of non-Hodgkin's lymphoma (NHL), defined as a malignant primary lymphoma occurring in the breast in the absence of previously detected lymphoma localizations. Our study aims to retrospectively evaluate the epidemiological, clinical, and imaging findings and therapeutic features of breast lymphomas in patients with primary lymphoma of the breast.

**Materials and methods:**

This is a retrospective study including 13 patients with primary non-Hodgkin's lymphoma of the breast treated at the Salah Azaiez Institute of Oncology from 2000 to 2019. This sample includes 1 case of follicular lymphoma, 2 cases of large T-cell lymphoma, and 10 cases of large B-cell lymphoma.

**Results:**

Patients included in the study were aged between 17 and 89 years (average age of 52.6 years). All patients were referred because of a lump in the breast, and only one patient consulted with inflammatory signs in the breast. The average clinical size of the tumor was 7.2 cm, with a maximum of 15 cm. Mammography showed an oval mass with circumscribed margins in the majority of cases. Ultrasound showed in most cases a hypoechoic irregular mass or multilobulated mass with irregular margins and hypervascular on color Doppler. Magnetic resonance imaging (MRI) was performed on only three patients and showed a spiculated lesion with polycyclic limits. Eight patients underwent surgery. In our study breast lymphomas involved 10 cases of large B-cell lymphoma, one case of follicular lymphoma, and two cases of large T-cell lymphoma. In this series, 11 patients had localized stages (I + II) at diagnosis, and 2 patients had disseminated stages (stage III) of primary breast lymphoma. Seven patients underwent chemotherapy treatment alone, and five had chemotherapy with radiotherapy. The median follow-up of our patients was 53 months, ranging from 1 to 177 months. Overall survival was 71% at 3 years and 51% at 5 years.

**Conclusion:**

Primary breast lymphoma is an uncommon type of breast malignancy. The optimal treatment modality is still in question because of the rarity of this disease. However, the use of combination therapy produces the most favorable results. Surgery is not yet recommended.

## Introduction

Primary breast lymphoma (PBL) is a rare presentation of non-Hodgkin's lymphoma (NHL); it is an uncommon neoplasm that was described for the first time in 1959 by Dobrotina. PBL is defined as a malignant primary lymphoma occurring in the breast in the absence of previously detected lymphoma localizations [1]. It is a rare entity accounting for less than 1–2% of all non-Hodgkin lymphomas and less than 0.5% of all malignant neoplasms of the breast [2]. Most primary breast lymphomas are of B-cell phenotype [3, 4, 6, 9]. It usually presents as a clinically palpable mass, the imaging characteristics are not specific, and they may sometimes mimic benign masses. The diagnosis is confirmed by biopsy. The treatment is based on a combination of chemotherapy and radiation therapy [9, 11].

### Patients and methods

This is a retrospective study including 13 patients with primary non-Hodgkin's lymphoma of the breast treated at the Salah Azaiez Institute of Oncology from 2000 to 2019. Patients were included based on the diagnostic criteria for primary lymphoma of the breast as described in 1972 by Wiseman and Liao and included an adequate tissue specimen available for diagnosis, no evidence of systemic lymphoma or history of extra-mammary lymphoma, excluding ipsilateral axillary lymph node involvement.

The clinicopathological data and the follow-up data of patients were collected through clinic visits. All the patients had a histopathological diagnosis of primary non-Hodgkin's lymphoma of the breast and had detailed and available clinical data. Survival data, details of lymphoma progression, and mortality from any cause were recorded. Tissue specimens were sampled by fine-needle biopsy, excision biopsy, lumpectomy, or mastectomy. Extensive immunohistochemical studies using a large panel of antibodies (B marker (CD20), T marker (CD3), CD30, CD45, CD15, vimentin, and CD10 were performed.

## Results

The 13 patients included in the study were aged between 17 and 89 years (average age: 52.6 years). The study population was predominantly female: nine patients were females, and three patients were males. In our study, three patients had a history of breast pathology; one had a history of an in situ ductal carcinoma, and two patients had a history of primary breast follicular lymphoma that became diffuse large cell lymphoma B after 2 years and 10 months. One patient had a family history of infiltrating ductal carcinoma. Four patients were menopausal. One of our patients, aged 30 years old, was five months pregnant at the time of the breast lymphoma diagnosis. All patients were referred because of a lump in the breast, and only one patient consulted with inflammatory signs in the breast. Tumors occurred mostly in the left breast [8 patients]; however, it was located in 5 cases on the right side and bilaterally in only one case. The average clinical size of the tumor was 7.2 cm; with a maximum of 15 cm. Axillary lymph nodes were palpated in 7 patients. The clinical examination revealed a supraclavicular lymphadenopathy in one case but it was reactional in the histological exam.

Mammography was performed in all cases; it showed an oval mass with circumscribed margins in the majority of cases, and only in three cases, it showed an opacity with indistinct margins. In one case it showed an irregular mass and a retraction and thickening of the superficial skin planes.

Ultrasound showed in most cases a hypoechoic irregular mass or multiloculated mass with irregular margin and hypervascular on color Doppler.

Magnetic resonance imaging (MRI) was performed on only three patients and showed a spiculated lesion with polycyclic limits. The mass was hypointense on T1-weighted MRI and isointense on T2-weighted MRI.

All patients underwent biopsies to confirm the diagnosis. Eight patients underwent surgery: six patients had a lumpectomy with axillary dissection, and two had a Patey intervention.

**Fig. 1:**
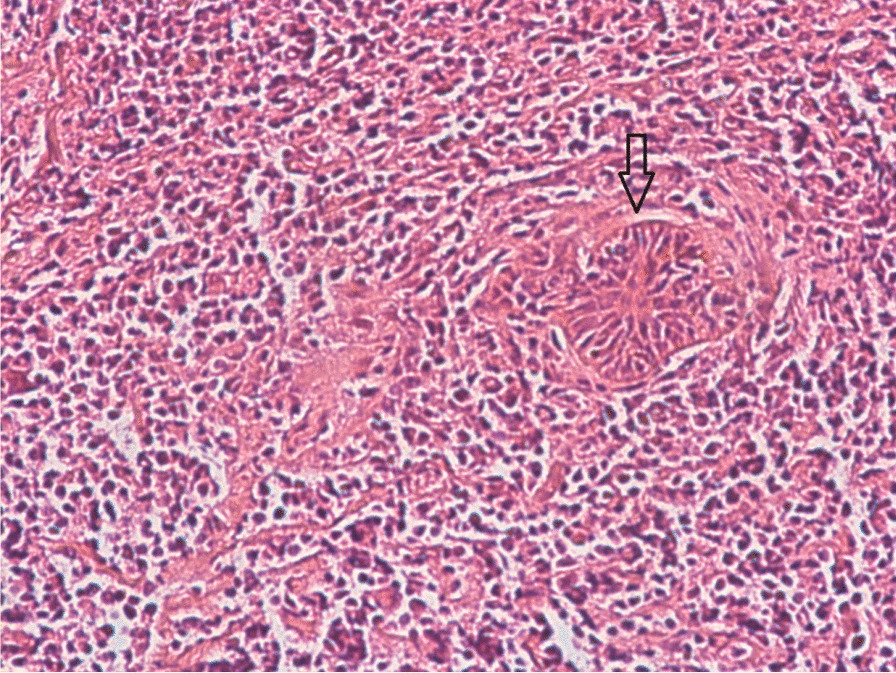
HE × 200 small cells diffusely infiltrating the mammary tissue (arrow next to a residual gland)

**Fig. 2:**
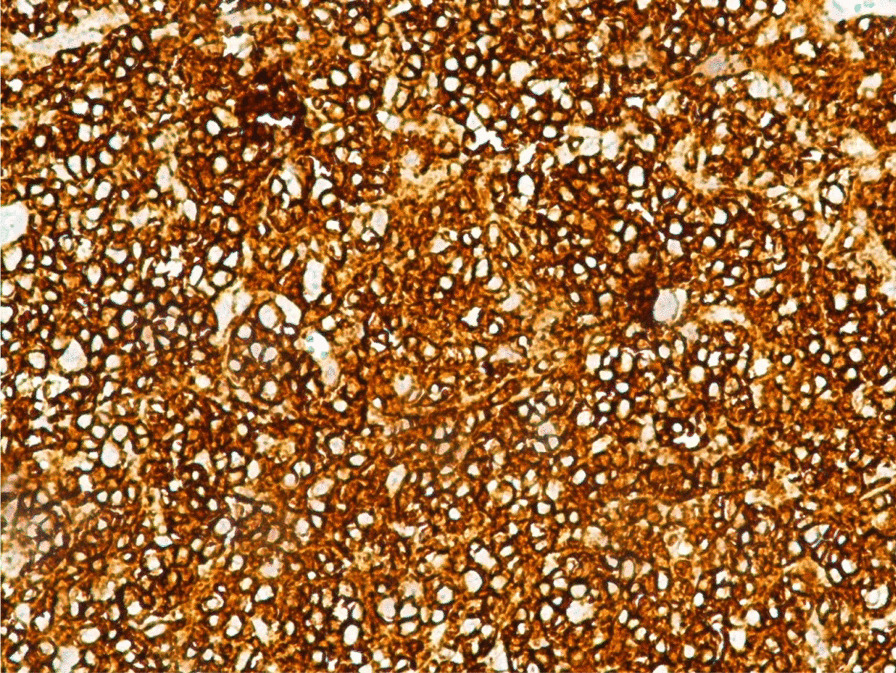
IHC × 200 showing diffuse and intense labeling of antiCD20

The immunohistochemical study (IHC) confirmed the diagnosis of non-Hodgkin's lymphoma (Fig. 1) and eliminated other diagnoses. A panel of antibodies was used: B marker (CD20), T marker (CD3), CD30, CD45, CD15, vimentin, and CD10. In 7 cases, the IHC results showed diffuse and intense labeling of antiCD20 (Fig. 2).

In our study, breast lymphomas involved 10 cases of large B-cell lymphoma, a case of follicular lymphoma, and two cases of large T-cell lymphoma. Eleven patients in our series had localized stages (I + II) at diagnosis, and two patients had disseminated stages (stage III) of primary breast lymphoma. Seven patients underwent chemotherapy treatment alone, and five had chemotherapy with radiotherapy. Surgery was performed in 8 cases. Five patients had a lumpectomy with axillary dissection, and 3 had a Patey-type surgery. For operated patients, they underwent neoadjuvant chemotherapy with 6 cycles of R-CHOP (Rituximab 375 mg/m^2^, Cyclophosphamide 750 mg/m^2^, Adriablastine 50 mg/m^2^, Vincristine 1.4 mg/m^2^ and Prednisone 60 mg/m^2^) every 3 weeks. The median follow-up of our patients was 53 months, ranging from 1 to 177 months. Overall survival was 71% at 3 years and 51% at 5 years.

## Discussion

Primary breast lymphoma is a rare localization of lymphomas. It is typically a B cell which accounts for up to 50% of all PBL [1]. A minority of cases are follicular lymphoma, mucosa-associated lymphoid tissue lymphoma, or Burkitt lymphoma [5, 10, 11].

There are some criteria for the diagnosis of PBLs defined by Wiseman and Liao in 1972, including (a) both mammary tissue and lymphomatous infiltrate present in close association in an adequate pathologic specimen; (b) no evidence of widespread lymphoma by standard staging techniques or preceding extra mammary lymphoma, except for ipsilateral axillary node involvement, if diagnosed simultaneously with the breast lymphoma [7, 11].

The Pathophysiology is still unclear, it is probably derived from mucosa-associated lymphoid tissue (MALT), lymphoid tissue adjacent to breast ducts and lobes, or even from intra-mammary lymph nodes [1, 7]. Also in the literature, some authors found an association between lymphoma and pregnancy, this finding suggests that hormonal disturbances are likely to play a role in inducing the proliferation of lymphoma [12].

PBL almost occurs in females; however, a few cases were reported in men. The most present age is between 60 and 65 years [13]. In our study, we found 3 men among 13 patients and the average age was 52.6 years (17–89 years). In some publications, PBL may occur at a younger age, which was the case of one of our patients who is aged 17 years; this presentation is associated with an aggressive prognosis.

The clinical presentation is similar to breast carcinoma. A single palpable and painless lump is the most common manifestation (61% of cases), but it can be multiple in some cases [4, 7]. Other cutaneous signs like nipple discharge, skin retraction, and local inflammatory signs are rarely seen. Ipsilateral axillary lymph nodes are reported in 13–50% of cases [13].

In the literature, about 12% of cases were found incidentally on mammography [7]. Particularly, in the case of lymphoma B cells, we can find systemic symptoms like fever, weight loss, and night sweats [11]. Usually, PBL involves the right breast (48%), but it can be bilateral in about 11% of cases [7, 11].

In the present experience, the most prominent initial signs were a tumor mass and rarely a mass with local inflammation (one case).

The diagnosis of PBL is based firstly on mammography and ultrasound. Indeed the imaging finding is non-specific.

Mammograms often show a solitary (75%) noncalcified and oval-shaped (50%) mass with circumscribed or indistinct margins (69%), spiculations are not seen in association with this malignancy. Rarely, global asymmetry may also be a presentation of PBL [1, 5, 11].

In Ultrasound, PBL appears usually as a hypoechoic (87%) solid oval or round mass with poorly defined contours and is usually hypervascular [5]. Posterior acoustic is present in 52–75% of the masses and hypervascularity in 55–64% [11]. Speculated margins or calcifications are rarely seen [1, 7].

Magnetic resonance imaging, if done, shows an isointense mass on T1 and hyperintense on T2 with homogeneous or heterogeneous enhancement [5, 11].

These radiology findings are similar to our study, in fact, mammography showed an oval mass with circumscribed margins in the majority of cases. Ultrasound showed in most cases a hypoechoic irregular mass or multilobulated mass with irregular margins and hypervascular on color Doppler. Magnetic resonance imaging (MRI) was performed in only three cases and showed a spiculated lesion with polycyclic limits. The mass was hypointense on T1-weighted MRI and isointense on T2-weighted MRI.

PET–CT is useful, especially in the evaluation of response to treatment. Also, it is valuable in the staging and follow-up of lymphoma patients. It may show the involvement of axillary nodes or other extranodal diseases in breast lymphoma [11]. None of our patients underwent a PET–CT because of the unavailability of this technique.

Like other breast lumps, the diagnosis is confirmed by histology. It is based on non-invasive techniques: fine needle aspiration, core biopsy, and excisional biopsy. Rapid histopathology during surgery is required to confirm the diagnosis and to avoid misdiagnosis [7, 16].

Treatment of PBL remains controversial, however, certain guidelines have been established; it may be based on a combination of surgery, radiotherapy, chemotherapy, and immunotherapy. However, there is no consensus on the best approach [7]. The guidelines depend on the histological subtype and stage. Indeed it is well established that the CHOP regimen is the standard treatment for primary diffuse large B cell lymphoma (DLBCL) of the breast and currently the introduction of targeted therapy [16].

In older literature, radical mastectomy was used for the treatment of PBL. But recent studies showed that it has no benefit, and may delay the start of chemotherapy. Moura *et al.* [7] found that surgery does not have any impact on survival or recurrence risk. It is only performed for diagnostic purposes in case of failure of non-invasive techniques.

Also, Luo *et al.* [16] found in their study that complete resection did not have a significantly improved prognosis. And he explained these findings by the fact that lymphoma is a hematologic malignancy, and its pathogenesis and progression will be different from that of solid tumors. So he concluded that systemic treatment may be more beneficial for tumor control than surgery.

Other authors demonstrated that surgery is associated with poor survival. In fact, Jeanner *et al.* [14] concluded that mastectomy was associated with poorer survival compared with systemic therapy and they thought that surgery should be limited to a biopsy to confirm correctly the diagnosis.

Also, Fruchart *et al.* [17] concluded in their study that included 19 patients that all patients undergoing mastectomy, either alone or in association with chemotherapy, died of their lymphoma. The reason seems related to the delay of systemic therapy. On the other hand, they concluded that mastectomy increases the risk of treatment failure and should be avoided.

On the contrary, Radkani *et al.* [4] found that the small number of patients in their series who underwent radical surgery had better local control. But this finding cannot be generalized because of the small number of patients included in this study. So surgery is not recommended, it should be limited to biopsy to obtain the correct histological diagnosis and in case of painful or hemorrhagic mass.

Currently, chemo-immunotherapy with consolidation radiation therapy is considered the mainstay in the treatment of PBL that has shown the most favorable results. Six cycles of CHOP (cyclophosphamide, doxorubicin, vincristine, and prednisone) or CHOP-like anthracycline-based chemotherapy combined with rituximab is now considered the standard treatment. This may be followed by radiation to the ipsilateral breast and regional nodes [7, 11].

Chemotherapy should be given at least 3 cycles because Luo *et al.* [16] demonstrated that among patients who received at least 3 cycles of chemotherapy, the 5-year OS was 40.7%, and the 5-year DFS was 33.6%. However, for patients who had < 3 cycles of chemotherapy, the 5-year OS and 5-year DFS were 10% (*P* < 0.01). Therefore, ≥ 3 cycles of chemotherapy were associated with an increased survival rate in patients with PBL.

Nevertheless, the choice of criteria for combination therapy is still controversial, most authors recommended these criteria: high-grade tumors, axillary lymph node involvement, and central nervous system (CNS) involvement in primary high-grade breast lymphomas have also been described [7].

Wong *et al.* [18] recommended that the modality chosen should depend on the histologic type of lymphoma and they think that systemic therapy should be reserved for high-grade disease.

However, Avilés *et al.* [19] reported in their prospective study that no prognostic factors can define treatment, and therefore, they recommend a combination of chemotherapy and radiation therapy for all patients with primary breast lymphoma [19]. Also, Joks *et al.* [1] indicated the use of combined therapy even in early stages because this protocol is more useful for patients with PBL.

In the series of Jeanner *et al.* [14], 61% of patients received radiotherapy, with or without chemotherapy. The median radiotherapy dose was 40 Gy (range 12–55 Gy) with a median daily dose of 2 Gy. The majority [8 of 10] of the patients with local relapses did not receive postoperative radiotherapy. Thus, radiotherapy to the breast or the thoracic wall had a statistically significant positive impact on local control, with 95% vs. 76% 5-year local control rate (*p* = 0.02). This confirms the central role of radiotherapy in PBL.

Luo *et al.* [16] showed that radiotherapy was not significantly associated with an improved 5-year OS and 5-year DFS. In this study, 12 patients received local radiotherapy with a dose range of 20–53 Gy (median 36.5 Gy).

Recently, combined modality treatment is the most useful in PBL. In fact, Jeanner *et al.* [14] demonstrated that combined therapy was associated with a favorable impact on local control (*p* = 0.03).

The same findings were found in the series of Aviles *et al.* [19] including 96 patients with PBL treated with three modalities: radiotherapy (*n* = 30), chemotherapy (n = 32), and combined modality treatment (*n* = 34). At 10 years, the overall survival was 50%, 50%, and 76%, respectively (*p* < 0.01). This confirms the positive impact of combined modality.

The role of rituximab is controversial, indeed some authors like Luo *et al.* [16] reported that rituximab did not affect the OS of patients with primary PBL of the breast. However, recent studies by Ludmir *et al.* have shown that rituximab has limited efficacy against recurrence in the breast and central nervous system, but significantly reduces the risk of systemic lymph node recurrence [21]. This study demonstrated also that radiotherapy did not improve patient prognosis [20].

There are many controversies about prognostic factors for patients with PBL. The most common factors are early stage (IE), the use of radiotherapy, and combined modality treatment. Other studies reported that tumor size and especially node status is the best single predictor of survival [1, 14].

Also, Luo *et al.* [16] found that Bcl-6 expression was associated with a better prognosis, but the size of the primary lymphoma in the breast was not significantly associated with the 5-year OS. Contrary to previous studies, Aviles *et al.* [19] did not find any significant prognostic factors that influenced response, event-free survival, or overall survival.

Thus, there are clear differences in prognosis factors identified in the literature and the management of high and low-grade BL. Indeed, we can conclude that tumor grade is a determinant factor for treatment strategies for high-grade PBL. In this case, treatment consists of a combination of anthracycline-based chemotherapy and rituximab followed by consolidative ipsilateral breast radiotherapy, which reduces the risk of local recurrence.

Even in our small study, the attitude is different depending on the authors. In fact, eight (8) patients underwent surgery, seven (7) received only chemotherapy, and radiotherapy was combined with chemotherapy in 5 cases.

In the literature, it is demonstrated that PBL is an aggressive tumor with high relapse rates, involving extranodal sites mainly the central nervous system and breast. And high central nervous system relapse rates in up to 20% of patients result in poor overall survival rates, so it is recommended to add central nervous system prophylaxis to systemic treatment in PBL [11].

## Conclusion

Primary breast lymphoma is a rare subset of extranodal lymphoma that behaves differently from nodal lymphomas, and the optimal treatment modality is still in question because of the small number of patients due to the rarity of this disease. Surgery is not yet recommended. However, the use of combination therapy produces the most favorable results. The prognosis is poor because of frequent relapses in the CNS. The identification of prognosis factors may help to indicate the adequate management of PBL.

## Data Availability

Data supporting our findings were taken from the patient’s folder.
